# Intubation of the Larynx

**Published:** 1888-06

**Authors:** John Dacre

**Affiliations:** Surgeon to the Bristol Hospital for Sick Children


THE BRISTOL

### Medico-Chirurgical Journal.

JUNE, 1888.

### INTUBATION OF THE LARYNX.

Read before the Bristol Medico-Chirurgical Society.

BY

JOHN DACRE, M.R.C.S.,

*Surgeon to the Bristol Hospital for Sick Children.*

I PROPOSE to bring forward for discussion a subject comparatively recently introduced into the domain of practical surgery—the operation of Intubation of the Larynx; and I do so because I think, from my own very limited experience of it, that it is one of distinct practical value, and one that demands a more extensive trial.

The operation as it at present exists is the one originated by Dr. Joseph O'Dwyer, of New York, and elaborated by him after a long series of experiments and considerable experience in its performance. Prior to his time various records are to be found of the introduction of tubes into the larynx for the relief of dyspnœa, but not until now does it appear to have been taken up as a recognised substitute for tracheotomy.

In 1801, Dessault demonstrated the fact that the larynx and trachea would tolerate the presence of a cannula, by accidentally passing an œsophageal tube through the nares into the larynx, instead of into the œsophagus. He did not discover his mistake until he began to inject the fluid that was intended for the stomach. He afterwards brought this observation to practical account in the treatment of a case of dyspnœa from œdema of the glottis. He passed a catheter into the larynx, relieved the dyspnœa, and allowed the instrument to remain for a day and a half; and the patient ultimately recovered.

Trousseau, in his article on the "Treatment of Diphtheria and Croup," reports that Dieffenbach practised catheterism of the larynx in 1839.

In 1854, Dr. Horace Green, of New York, in a paper read before the New York Academy of Medicine, stated that he was able to introduce a sponge probang into the larynx and trachea, and could pass a flexible tube into either bronchus, which could be used either to relieve dyspnœa simply, or for the purpose of injecting tubercular cavities in the lung.

Bouchut, in 1858, before the Academy of Medicine at Paris, introduced an operation practised by him, which he styled "Tubage of the Larynx."

He used short cylindrical tubes hardly an inch in length, and introduced them into the aperture of the glottis by means of a sound. A silk thread attached to the tube prevented it from being inspired or swallowed, and enabled it to be withdrawn when necessary. He appears, however, to have brought the operation forward before he was able to give satisfactory proof of its practical utility; for out of seven cases which he reported, five died, and the other two only recovered after tracheotomy had been performed.

A committee, with Trousseau at its head, was appointed to investigate the subject; and the conclusions that were arrived at after discussion seem to have so far ridiculed and condemned the operation, that Bouchut never again brought it forward, and it soon sank into oblivion.

Macewen, of Glasgow, published some cases in 1880, showing the possibility of relieving urgent dyspnœa by the introduction of a catheter into the larynx.

Other surgeons have, at different times, reported cases of tubage of the larynx; but none of them have elaborated their methods to anything like perfection, and consequently they have never become of any general value.

To-day, however, thanks to the zealous and untiring labours of O'Dwyer, intubation of the larynx is a recognised surgical procedure. He was first led to turn his thoughts in this direction, I believe, from the ill-looking statistics of his own tracheotomies; and, without any knowledge of what had been previously accomplished, he set to work in the hope of discovering some satisfactoy method of introducing a tube by the mouth.

The mortuary of the Children's Hospital in New York afforded him the opportunities for performing his experiments; and after much investigation and many devices, he has at last arrived at the conclusion that the instruments which are at present made are, at any rate, satisfactory for the purpose for which they are intended; but, at the same time, he expresses a hope that, in details, they may yet be improved upon.

The instruments as usually sent out consist of a set of five tubes, varying in length from 1¼ to 2½ inches, to suit individual cases; of an introducer, and an extractor.

Each tube is perfectly straight, and of sufficient weight to remain steadily in position. The upper extremity is flanged, to rest on the margin of the glottis, and is perforated at the anterior part by a small aperture, through which can be passed a fine string of silk, to be held in the hand during the operation. The lower extremity is elliptical in section, with the edges neatly rounded off. About its middle the tube is bulged out—a device which prevents the too ready expulsion during coughing. The interior of the tube is filled by a movable obturator, which can be screwed on to the introducing instrument above, and, projecting beyond the lower aperture below, serves to make the instrument a probe-pointed one when being inserted, thereby obviating the risk of scraping the surface of the laryngeal mucous membrane by the unprotected edge of the tube.

A small scale usually accompanies the tubes, marked with numbers that indicate the years for which the corresponding tubes are suitable.

The introducer is about the size and shape of a No. 4 silver catheter, provided with a handle; and the obturators are screwed on to a thread at its extremity. A sliding rod, worked from the handle, serves to push off the tube when it is fairly in position.

The instrument called the extractor is of ingenious device. The blades are passed *closed* into the opening of the tube, and by pressure with the thumb on a small lever they are made to separate, and to hold the tube sufficiently firmly for extraction.

To these instruments must be added a good reliable gag, working from the left side.

The following is the method of introducing the tube, which is done without an anæsthetic: The child is wrapped up in a blanket, in order to restrain the arms by the side, and is held upright as firmly as possible on the knees of a nurse or other reliable person. The nurse should sit in a straight-backed chair, and hold the child with its back firmly against her chest. An assistant from behind then holds the head of the patient, depressing the chin slightly, and at the same time taking charge of the gag, which is inserted in the left angle of the mouth and opened widely.

The operator then—sitting squarely before the child—passes his left index finger along the tongue and into the pharynx. Then, by bringing the finger forwards, the epiglottis is lifted up, and the aperture of the larynx should be distinctly felt.

The handle of the introducer is held close to the patient's chest at the beginning of the operation, and is rapidly elevated as the tube approaches the glottis. Guided by the left forefinger, the tube sinks into the aperture, and is gently pushed downwards.

When in position, the head of the tube is held by the finger, and by pushing the sliding rod of the instrument, the obturator is quickly removed. The left finger is then withdrawn, and the right hand still holds the string which is attached to the tube.

If the tube be in the larynx, and there be no complication, the child will cough honestly, it will expectorate freely, and the breathing will have lost all stridor.

If the symptoms be aggravated, the tube has most likely been passed into the œsophagus. This being so, it should of course be immediately withdrawn by means of the string, and the operation repeated.

It is important that the introduction should be effected quickly, as respiration is practically suspended for the time being; and it is better, therefore, to make several fruitless attempts rather than one prolonged one.

Until sufficient confidence has been gained by experience, it is well to leave the string attached to the tube and looped around the ear, as the operation for the extraction of the tube is not so easily accomplished. Should the string be allowed to remain, the child should be closely watched, to prevent it either clutching it with its hand, or lifting the tube out of place by the movements of the tongue.

For the purpose of removing the tube by the extractor, the child is held in a similar position as for introduction; the gag is introduced; and the points of the extractor, guided by the left forefinger, are passed into the opening of the tube. Firm and continuous pressure on the lever above the handle is then made with the thumb, and the expanded points give sufficient hold on the tube for removal.

Just as there are dangers in the performance of tracheotomy, so there are dangers in the performance of intubation; and before discussing the relative merits of the two operations, it is only right that these faults should be fairly pointed out.

The accidents liable to occur from intubation may be thus enumerated:

1. Asphyxia may be produced by a too prolonged attempt at introduction.
2. False passages may be made by the use of too much force.

3. Membrane may be pushed into the trachea before the incoming tube.
4. Asphyxia from blocking of the tube.
5. Asphyxia from œdema crowding over the top of the tube.
6. Coughing out of the tube, followed by an immediate return of urgent symptoms.
7. Ulceration of larynx or trachea from pressure.
8. The inspiration of food into the tube, setting up pneumonia.

The first and second of these accidents may distinctly be placed under the heading of avoidable ones, and are not likely to occur in the hands of any one possessed of ordinary skill and care.

The third accident is to a great extent prevented by having the lower end of the tube made probe-pointed by the projecting obturator; and, indeed, does not occur by any means so frequently as would naturally be supposed. In fact, only five cases can I find amongst the many that are reported in which it happened. Removal of the tube in four of these cases was followed at once by the expectoration of the consolidated membrane, and in one only was death attributed to its occurrence.

O'Dwyer says that the fourth accident can never occur; for immediately there is the slightest resistance to the expired air, the tube is expelled. In a case of my own, however, secondary dyspnœa supervened from blocking of the tube. Yet, owing to extension of the disease to a lower level, the expiratory efforts were too feeble to expel it; and tracheotomy failed to gain an opening below the obstruction.

The same remarks would apply to the obstruction caused by inflammatory swelling of the glottis around the upper opening of the tube.

The onset of urgent dyspnœa after accidental expulsion of the tube undoubtedly does happen sometimes, and cases are recorded in which a fatal issue has resulted before the surgeon could arrive in order to replace the instrument; although, to the credit of the operation, it must be owned that such unfortunate terminations are few and far between. With a properly-selected tube well in position, and the string detached, the danger of expulsion is reduced to a minimum, and it by no means necessarily follows that a paroxysm of dyspnœa will occur if it should be expelled.

Ulceration from pressure is of course just as likely to occur from an unsuitable intubation cannula as from an ill-fitting tracheotomy tube; so that with a little care the risk is obviated.

The inspiration of food into the air passages certainly gives cause for anxiety in a good many cases, according to the records of those with the largest share of experience, but personally I have not met with the difficulty; if I had, I should have relied upon nutrient enemata to supply the requisite nourishment.

In spite, however, of these unfavourable contingencies that may arise from intubation, the statistics of operations already recorded compare very favourably with those of tracheotomy. The percentage of recoveries after each operation is $26\frac{2}{5}$. Intubation has decidedly an advantage in patients under the age of five years; but above that age tracheotomy has the better record.

The following statistical table, showing the percentage of recoveries after each operation, is from a paper by

Max Stern, of Philadelphia, read at the International Medical Congress at Washington:

| | | | | | Intubation. | Tracheotomy. |
|---|---|---|---|---|---|---|
| Under | 2 | years | ... | ... | 15 per cent. | 3 per cent. |
| Between | 2 | ,, | and | 2½ ... | 24 ,, | 12 ,, |
| ,, | 2½ | ,, | ,, | 3½ ... | 28 7/10 ,, | 17 ,, |
| ,, | 3½ | ,, | ,, | 4½ ... | 33 7/10 ,, | 30 ,, |
| ,, | 4½ | ,, | ,, | 5½ ... | 28 3/10 ,, | 35 ,, |
| Above | 5½ | ,, | ... | ... | 37 3/10 ,, | 39½ ,, |

To such a good record intubation presents certain advantages over tracheotomy:

1. The tube is worn much more comfortably by the patient.
2. The air, having to enter by the natural channels, is warmed as in normal respiration.
3. Coughing, and therefore expectoration, are more effectual.

And last, but by no means least—an advantage which is especially noticeable in private practice—the operation is not so formidable a one as tracheotomy. It can therefore be urged much earlier than the more desperate operation would be, and the consent of friends can be more readily obtained for its performance; and any method that will give equal relief, at the same time dispensing with the painful incidents of a tracheotomy in private practice, should indeed be welcome.

Such are the main data of intubation of the larynx. Whether the operation is being much practised in this country, I cannot say; but in America it has long since passed the stage of probation, and has now gained so large a share of popularity as to be practised on a scale so extensive as would only be justified by satisfactory results.

Good results undoubtedly have been attained—so encouraging, indeed, as to warrant the assumption that it may still be more successful in the future. A wide field of trial is required to elicit suggestions that will reduce the dangers to a minimum; and when that stage is reached, I certainly think we shall have at our command an operation that will possess all the advantages that tracheotomy now can offer us—one easy of accomplishment, and one that will be of unquestionable service in saving many lives.

